# Minimally invasive reduction of vertically displaced sacral fracture without use of traction table

**DOI:** 10.1007/s10195-011-0132-4

**Published:** 2011-02-24

**Authors:** Alberto Nicodemo, Claudio Cuocolo, Marcello Capella, Martino Deregibus, Alessandro Massè

**Affiliations:** Department of Orthopaedics and Traumatology, San Luigi Gonzaga Hospital, University of Turin, Regione Gonzole 10, 10043 Orbassano, TO Italy

**Keywords:** Sacral fractures, Minimally invasive reduction, Pelvic instability, Ilio-sacral screw fixation, Slide-insertion plate

## Abstract

**Background:**

Treatment of vertically displaced sacral fracture can be difficult even for the expert traumatologist. Traditional reduction methods can show some limitations; we suggest a minimally invasive technique, which could be effective, tissue sparing and economic in terms of equipment needed.

**Materials and methods:**

Our retrospective study included 11 patients with average age of 40.2 years (range 24–59 years), with type C pelvic ring disruption with monolateral sacral fracture (C1.3), who underwent surgical treatment from April 2007 to March 2008 using the minimally invasive technique. Radiographic examination, using Matta’s criteria, was carried out pre-operatively, post-operatively and at least at 1 year after surgery. All patients were functionally evaluated using Majeed’s grading scale with mean follow-up time of 18.9 months (range 14–25 months).

**Results:**

Pre-operative displacements averaged 10.8 mm (range 7–21 mm); post-operative displacements averaged 5.4 mm (range 3–12 mm), with excellent or good reduction in 91% of cases. No major complications occurred. On functional evaluation, 82% of patients obtained good or excellent results.

**Conclusion:**

The minimally invasive reduction technique, requiring a limited surgical approach and a standard radiolucent table, is in our experience a satisfactory procedure for management of monolateral vertically displaced sacral fracture.

## Introduction

A lot has been written about minimally invasive stabilization of sacral fractures with percutaneous ilio-sacral screws [1–5] or posterior sacral plates [6], but few articles give sufficient details on the reduction methods. The achievement of satisfactory reduction is the first hot point in the treatment of pelvic ring disruption [7]. Despite its importance, this step may be a real challenge even for the expert pelvic surgeon, because of several issues including the frequent finding of multi-planar and rotational displacement components even in so-called vertically displaced fracture. The classic traction method is often insufficient to deal with these difficulties, because of its exclusively axial effect, the large force required and the need to fix the intact hemi-pelvis in a strong and safe manner. Only some of these problems seem to be solved by use of special pelvic frames [8, 9], and moreover, radiolucent traction tables are expensive devices, available only in few hospitals.

Otherwise, open reduction techniques can provide good results, but are expensive for these patients in terms of blood loss [7, 10]. Furthermore, surgical access has to be achieved via a skin area often damaged by trauma and in zones that are subject to bedsores.

The aim of this study is radiological and functional evaluation of a minimally invasive reduction technique for treatment of sacral fracture, which could be effective, tissue sparing and economic in terms of equipment needed.

## Materials and methods

From November 2002 to March 2009, 82 patients suffering from sacral fracture were surgically treated at our institution. Among these, 51 patients presented 61-C1.3 fracture according to the Orthopaedic Trauma Association (OTA) [11].

The method of reduction was predominantly closed with traction (28 cases), then open with posterior surgical access. Starting from April 2007, we began to perform the minimally invasive technique described herein.

For this retrospective study, inclusion criteria were: 61-C1.3 fracture pattern according to the OTA [11] (type C pelvic ring disruption with monolateral sacral fracture), availability of complete clinical and radiological documentation and a minimum 12-month follow-up time. Exclusion criteria were presence of cognitive deficits, major head trauma, neurologic deficits related to extrapelvic lesions, major injuries to the upper and lower limbs, open fractures and pathological fractures. Moreover, patients with significantly impaired mobility or pain during gait already present before the trauma were excluded.

We finally included in this study 11 patients with average age of 40.2 years (range 24–59 years), who were referred to our institution from April 2007 to March 2008 and surgically treated by the same surgeons (A.M., A.N.) using the technique indicated in Table 1.

**Table 1:** Patients’ data, procedures and outcomes

Case	Type of trauma	Age (years)	Time from trauma to surgery (days)	Denis	Follow-up time (months)	Majeed score	Displacement (mm)			Posterior fixation	Anterior fixation	Associated lesions
							Pre-surgical	Post-surgical	Follow-up			
1	Industrial accident	24	7	1	18	46	21	6	6	Two IS screws, tension band	Ex-fix	Urethral tear
2	Industrial accident	59	10	1	19	88	7	3	4	One IS screw	Four-hole plate	L4-L5 fracture (internal fixation)
3	Sports injury	35	7	1	17	99	14	4	4	Two IS screws	Ex-fix	
4	Car accident	57	13	1	25	81	8	4	4	Two IS screws	Six-hole plate, ex-fix	
5	Sports injury	37	2	2	15	98	9	6	5	One IS screw	None	Two-column acetabular fracture
6	Car accident	28	2	2	22	81	8	5	5	One IS screw	Ex-fix	
7	Motorbike accident	39	6	2	24	82	7	3	4	One IS screw	Four-hole plate	Bladder tear
8	Motorbike accident	43	2	2	18	71	8	6	6	One IS screw	Four-hole plate, ex-fix	
9	Industrial accident	33	4	2	14	94	12	7	8	One IS screw	Ex-fix	Splenic rupture
10	Sports injury	48	10	2	18	84	8	4	4	One IS screw	Four-hole plate	L1 fracture (conservative)
11	Sports injury	37	16	3	18	66	17	12	13	Two IS screws	Four-hole plate	Tibial plateau fracture, tibial shaft fracture
	Mean	40.0	7.18	–	18.91	80.91	10.82	5.45	5.73	–	–	–
	SD	11.02	4.68	–	3.45	15.16	4.66	2.54	2.72	–	–	–

*SD* standard deviation, *IS* ilio-sacral

The trauma was caused by road accident in 36% of cases, by sports injury in 36% and by industrial accident in 28%.

Seven patients had associated injuries, six of which required surgery: two urological lesions, one spleen rupture, one two-column acetabular fracture, one L4-L5 vertebral fracture and one bilateral lower limb fracture.

**Fig. 1:**
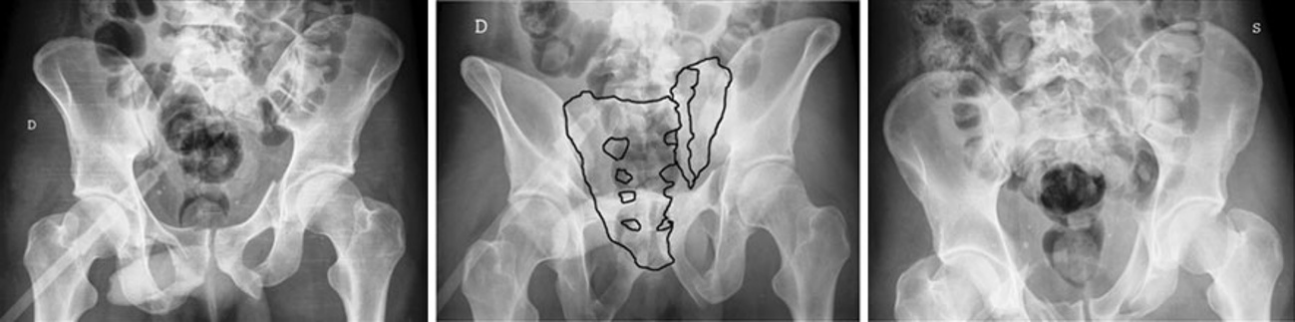
Illustrative case: pre-operative radiograms (antero-posterior, outlet, inlet) showing a multi-planar displacement with maximum value of 21 mm

Every patient was submitted to accurate clinical examination and pre-operative imaging planning, including at least antero-posterior (AP) radiogram of the pelvis, inlet and outlet views (Fig. 1) and a computed tomography scan. Every fracture was classified according to Denis classification [12]; the most common were type II (54%) and type I (36%). Pre-operative displacements were measured to the nearest millimetre as the maximum point-to-point distance between the fragments of the sacral fracture on each of the three views of the pelvis; all displacements were recorded.

The patients were brought to the operating room as soon as permitted by general health conditions, since it is well known that reduction becomes progressively more difficult with time; mean trauma to surgery time was 7.18 days [range 2–16 days, standard deviation (SD) 4.68 days]. Each fracture was reduced by the minimally invasive approach described below. Fixation of the posterior pelvic ring was achieved by one cannulated ilio-sacral screw in 64%, by two ilio-sacral screws in 28% and by two ilio-sacral screws plus tension-band plating in one case; all screws were placed in the body of the first sacral segment. Fixation of the anterior pelvic ring was achieved with a symphyseal plate in 36% and with an external anterior fixator in 36%; in two cases they were used together, while in one case no anterior fixation was performed.

**Fig. 2:**
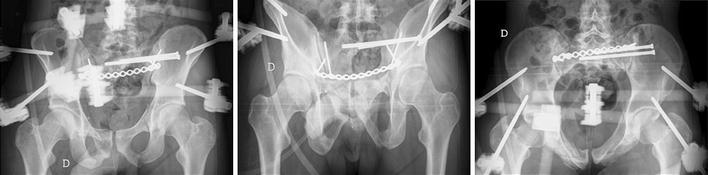
Illustrative case: post-operative radiograms. Fixation is achieved by two ilio-sacral screws, a posterior tension-band plate and anterior external fixation. Maximum residual displacement was 6 mm

After surgery, AP, inlet and outlet view radiograms of the pelvis were taken for every patient and post-operative displacements were measured on all three views (Fig. 2); we considered the highest value as an index of quality of reduction according to Matta’s criteria [7].

Patients were not allowed weight-bearing for 60 days. The external fixator, when used, was removed 2 months after its placement. They were then directed towards walking rehabilitation, with complete weight-bearing allowed 90 days after injury. Clinical and radiographic examination was carried out at least at 1, 2, 3, 6 and 12 months after surgery.

Finally, all patients were evaluated functionally at least 1 year after surgery using Majeed’s grading scale for pelvic fracture [13], with mean follow-up time of 18.9 months (range 14–25 months, SD 3.45 months). The study conforms to the 1964 Declaration of Helsinki as revised in 2000 and was approved by our institutional ethical committee. All enrolled patients provided informed consent.

### Surgical technique

The patient is under general anaesthesia, in prone position, on a standard radiolucent orthopaedic table. The patient position is exactly symmetrical, with forward tilting of the pelvis achieved by insertion of a thoraco-pelvic support, the knees being flexed at about 30° to release the sciatic nerves and sacral roots. The C-arm fluoroscope is placed on the uninjured side of the patient, and adequate image rendering is verified before starting the operation. The patient’s body has to be placed as caudal as possible, to avoid impingement of the C-arm and table’s pedestal; this can be achieved by assembling two standard leg attachments. The posterior pelvis is then prepped and draped in usual fashion.

**Fig. 3:**
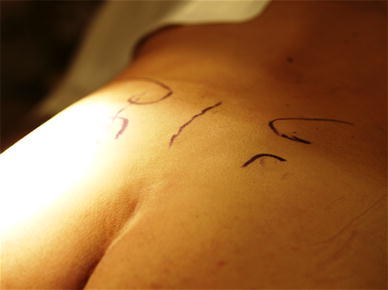
Bony landmarks drawn on the skin. The PSIS and the sciatic notch are individuated bilaterally by palpation. In the *middle*, the sacral spinous line

**Fig. 4:**
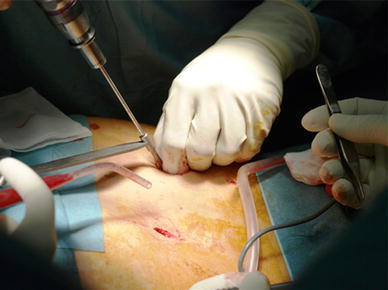
Surgical access to the PSIS (*right side*) and 3.2-mm drilling to insert the 4.5-mm screw (*left side*)

**Fig. 5:**
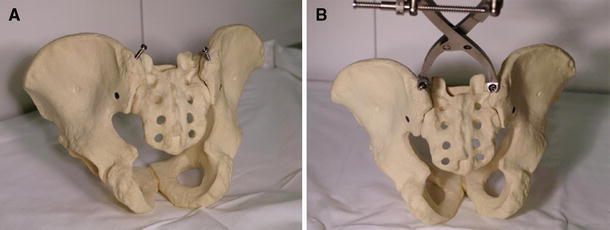
**a** Anatomic model showing typical starting and direction of the PSIS drilling, angled approximately 40° laterally and 10° cranially. **b** Connection of the Jungbluth clamp to the screws, which are later tightened to perform the fracture reduction

The posterior superior iliac spines (PSIS) are individuated bilaterally by palpation, and two incisions (about 1.2 inches each) are performed just lateral to them (Fig. 3); then a 3.2-mm drill is used to insert a cortical 4.5 screw in each side (Fig. 4). The drill is started on the posterior superior iliac spine, angling lateral approximately 40° in relation to the sagittal plane and slightly cranially to achieve placement in the direction of the iliac wing (Fig. 5a).

**Fig. 6:**
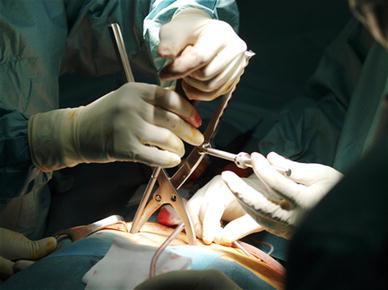
When the reduction seems satisfactory, the Jungbluth clamp is tightened and the result is checked on the three standard fluoroscopy views

Next, a large Jungbluth reduction clamp (Matta Pelvic System–Stryker Trauma AG, Bohnackerweg 1, CH-2545 Selzach, Switzerland) is connected to these screws (Fig. 5b). If an important rotational dislocation is detected, a standard 6.0-mm Shanz screw placed proximal to the 4.5 screw in the iliac wing can be used as a joystick. When the fracture displacement is satisfactory reduced, the clamp is tightened to maintain the reduction (Fig. 6). The reduction is checked by fluoroscopy in AP, inlet and outlet views on the posterior ring, and when obtained, it is common to see also the anterior part of the ring in a reduced position.

Fixation is usually achieved with ilio-sacral stainless-steel 6.5- or 8.0-mm cannulated screws (Asnis III; Stryker Orthopaedics, 325 Corporate Drive, Mahwah, NJ 07430, USA) under fluoroscopic control [1–5]. Most times, two screws are placed in the first sacral segment, even if it is possible to place one screw in S1 and one in S2, or a single screw may be used if the area of safe placement is limited [14]. At the end, screw position has to be confirmed on antero-posterior, inlet and outlet views [15–19]. In very unstable disruptions, and whenever possible and safe, use of a trans-sacral screw is suggested, as this type of fixation seems to provide greater stability [20].

After removal of the Jungbluth clamp, it is possible to implant a slide-insertion posterior plate through the same incisions [21, 22] to improve fracture stabilization. This is particularly suggested in case of large displacement, long trauma to intervention time (15 days or more) or poor bone quality.

After fixation of the posterior pelvic ring and closure of wounds, the patient is placed in supine position for anterior pelvic ring fixation, if necessary, by internal or external devices [23–25].

## Results

Pre-operative displacements averaged 10.8 mm (range 7–21 mm, SD 4.66 mm) (Table 1). The largest displacement was seen on the 40° caudal view in 63% of cases. Post-operative displacements averaged 5.4 mm (range 3–12 mm, SD 2.54 mm). Using the grading criteria described by Matta [7], there were five excellent (45.5%), five good (45.5%), one fair and no poor reductions. All patients healed, with average displacement at 1-year follow-up of 5.73 mm (range 4–13 mm, SD 2.72 mm). The improvement obtained with surgery was strongly significant (paired-sample *t*-test: *P* < 0.0009), while the difference between post-operative and follow-up displacement was nonsignificant (paired-sample *t*-test: *P* = 0.192).

**Fig. 7:**
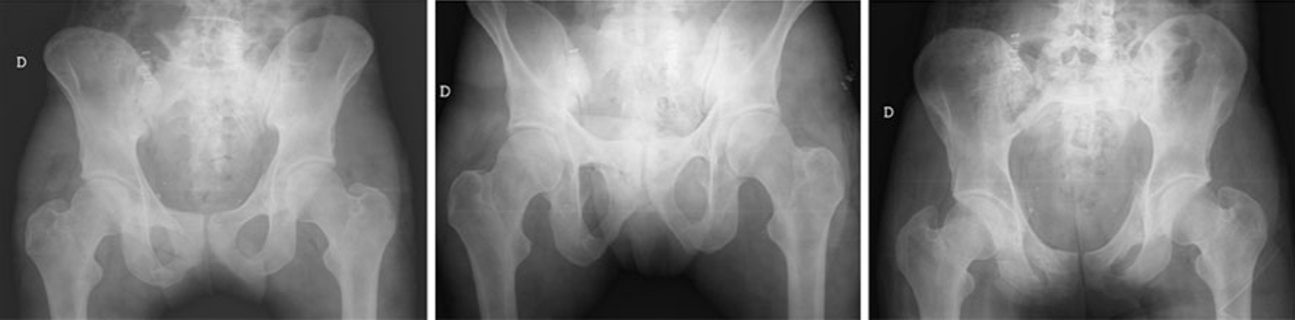
Illustrative case: after removal of external and internal devices, performed about 9 months after the accident, there was no sign of fracture displacement

There were no operative complications; regarding nonsurgical peri-operative complications, there was one urinary tract infection. In one case, aseptic loosening of the symphyseal plate was detected at 2-month follow-up; the hardware was removed 10 days later, without need for further fixation. In two cases, ilio-sacral screw removal, due to screw-related pain, was performed 9 and 11 months after surgery. In one further case, treated with two ilio-sacral screws and a sacral plate, the hardware was removed 9 months after the accident (Fig. 7) because it generated discomfort while the patient was using sports equipment (scuba diving gear with air cylinder). We do not consider this to be a device-related complication but rather a sign of surgical success.

On functional evaluation, performed using the Majeed score system, 82% of patients obtained good or excellent results, with only one fair and one poor result; the average score was 80.91 points (range 46–99 points, SD 15.16 points). All but one patients returned to work, but the majority of them complained of reduced performance.

A 23-year-old male affected by urethral disruption, treated with anterograde and retrograde endoscopic repair by a specialized urologic team, complained of reduced sexual function; at 12-month follow-up he reported fair improvement but not complete remission yet. In this case it is difficult to state whether this reflects a real neurologic issue, and whether it could be related to damage to periurethral neural fibres or sacral roots. We registered slight erectile dysfunction in two further males without urologic tears, who had almost complete recovery at 12-month follow-up. There were no further perineal deficits.

There were no major lower limb neurologic impairments, but in two cases we observed transitory lateral femoral cutaneous nerve hypoestesy, probably related to anterior external fixation; in one case recovery was complete within 6 months, in another case a partial sensory deficit remained at 12-month follow-up.

## Discussion

Reduction of vertically displaced sacral fracture can be a difficult challenge for the pelvic surgeon. Open techniques can obtain good reduction but are costly for the patient in terms of blood loss and soft tissue damage. Tornetta and Matta [7] stated that 10 mm is an acceptable reduction for injury to the posterior pelvic ring, as they suggested that greater anatomic reduction of posterior injuries did not result in less posterior pain; those authors, performing open reduction and internal fixation of the injured pelvis, reported excellent or good results in 95% of 107 patients with 38 Bucholz type II (type B) injuries and 69 Bucholz type III (type C) injuries, with 1 case of loss of reduction and 3 cases of deep infection. Van den Bosch et al. [26] evaluated 37 patients (16 type B, 21 type C) treated with internal fixation, obtaining a mean score of 78.6 of 100 on Majeed functional evaluation. Lindahl and Hirvensalo [27] obtained excellent-good radiographic results in 90% of 101 patients treated with open reduction; the overall functional results, measured with a modified version [28] of the original Majeed scoring system [13], were good or excellent in 83% of patients.

Percutaneous technique is becoming increasingly popular because it can reduce wound-related problems and blood loss [1, 2, 4, 15]. On the other hand, closed reduction using vertical traction can sometimes be insufficient, even with the adjunct of dedicated pelvic frames [8, 9]; moreover, it needs a radiolucent traction table. Routt and Simonian [4] defined reductions that showed less than 1 cm residual displacement in any plane as acceptable. They obtained only 3 malreductions among 60 sacral fractures treated with closed reduction by manipulation and traction method, but reported 5 cases of failure of fixation and 2 cases of non-union.

Schweitzer et al. [2], revising 71 pelvic ring fractures (10 type B, 61 type C) treated with closed reduction and percutaneous screw fixation, obtained 69 satisfactory reductions and 62 good or excellent functional results according to the Majeed scoring system [13]. Nevertheless, they reported a 9% rate of surgical-related complications, a rate similar to previous reports regarding this technique [5].

Minimally invasive transiliac plate osteosynthesis [21, 22] has been recently described, and its early results appear encouraging. However, this technique has usually been associated with the closed reduction by traction method.

The method described herein can obtain good fracture reduction with a limited surgical approach and with a standard radiolucent table; it is particularly indicated in simple, monolateral vertical sacral fractures (61-C1.3). Otherwise, we suggest reduction by traditional techniques for bilateral or complex sacral fractures, because of the extreme posterior instability, as well as for those disruptions which require direct vision of the fracture site.

The position of the patient allows traditional open exposure if minimally invasive reduction fails and allows internal fixation of the posterior ring and optimal placement of percutaneous ilio-sacral screws.

Using this technique we achieved good to excellent reductions in 91% of patients, coherent with most of the studies regarding sacral fractures which can be found in literature [2, 7, 27]. In only one patient was a fair result (>10 mm) obtained; he presented wide pre-operative displacement, and because of the long trauma to surgery time, interposition of soft tissue and fibrous callus formation prevented a better result; nevertheless, we decided not to perform open reduction because the skin and subcutaneous tissues were compromised. Unfortunately, he reported one of the lower functional scores at 1-year follow-up. We suggest that open reduction should be preferred, whenever possible, where the trauma to surgery time is extended beyond 15 days.

The main limitations of this study are the small sample size and the short follow-up; furthermore, its strength is blunted by all the implications of its retrospective design and the lack of a control group.

In conclusion, we found a minimally invasive reduction technique to be a satisfactory procedure for management of vertically displaced sacral fracture. Axial traction remains a good method, and more cases need to be operated using this technique to confirm its effectiveness in terms of reduction and determine possible complications.
